# Multi-Organ Adverse Reaction to Two Hypomethylating Agents: A Challenge in High-Risk Myelodysplastic Syndrome Treatment

**DOI:** 10.3390/hematolrep17030029

**Published:** 2025-05-30

**Authors:** Sofia Brites Alves, Francesca Pierdomenico

**Affiliations:** Hematology Department, Instituto Português de Oncologia Francisco Gentil (IPO), 1099-023 Lisbon, Portugal

**Keywords:** myelodysplastic syndrome, hypomethylating agent, azacitidine, decitabine, hypersensitivity, hemato-oncological patients, adverse drug reaction, drug-induced cutaneous reaction, drug-induced pulmonary toxicity

## Abstract

**Background and Clinical Significance:** Intermediate- to high-risk Myelodysplastic Syndrome (MDS), according to the Revised International Prognostic Scoring System (IPSS-M), confers a high risk of progression into acute myeloid leukemia. Treatment with hypomethylating agents, including azacitidine and decitabine, represents the current standard of care. In eligible patients, hypomethylating agents are used as a bridge for allogeneic stem cell transplantation, currently the only curative approach in these malignancies. The most common side effects of hypomethylating agents are myelosuppression, cutaneous injection site reactions (when azacitidine is given subcutaneously), and gastrointestinal symptoms. Uncommon, disabling, and long-lasting side effects represent a threat to effective treatment in this group of patients. **Case Presentation**: We describe the case of a 49-year-old male patient with IPSS-M intermediate-risk MDS, intended to receive first-line treatment with azacitidine followed by allogeneic stem cell transplantation. The first, late-onset azacitidine reaction was observed 48 h after the first exposure, with cutaneous and respiratory toxicity, followed by the late-onset recurrence of symptoms after azacitidine withdrawal and decitabine introduction. **Conclusions**: This case highlights atypical, disabling, and long-lasting drug reactions to two hypomethylating agents, with the persistence of hypersensitivity manifestations months after medication withdrawal.

## 1. Background and Clinical Significance

Myelodysplastic Syndromes (MDSs), recently renamed “Myelodysplastic Neoplasms” by the World Health Organization, are hematologic malignancies characterized by hypercellular bone marrow, morphological dysplasia due to ineffective hematopoiesis, and the consequent development of cytopenias with a risk of progression to acute myeloid leukemia (AML). The Molecular International Prognostic Scoring System (IPSS-M) identifies subtypes with a higher risk of developing AML. Depending on the IPSS-M results, patients might be treated with a hypomethylating agent (HA) such as azacitidine (AZA) or decitabine, with or without the aim to proceed to consolidation with allogeneic hematopoietic stem cell transplantation (allo-HSCT), which is, to date, the only potential curative treatment [1].

HAs are well-known drugs that have been used for two decades either as monotherapy or in combination for the treatment of hematologic malignancies including MDS, chronic myelomonocytic leukemia, and AML. The most common side effects are myelosuppression with consequent infection risks and gastrointestinal symptoms. Inflammation at the injection site is also a frequent side effect of AZA, as it is primarily administered subcutaneously.

Hypersensitivity reactions are infrequent, described only in 0,25% of cases in pivotal studies, and they include cutaneous rash and lung disease in the form of interstitial lung disease (ILD) or pneumonitis induced by AZA, which are sporadically reported in some studies [2,3,4]. Some pivotal phase III comparative studies (AZA 001, CALGB 9221) did not report on interstitial pneumonitis or ILD; however, the AZA 001 trial reported a case of pulmonary fibrosis. 

Y. Shimoda-Komatsu et al. [5] proposed different patterns for AZA-induced cutaneous reactions after reviewing 21 such cases: erythematous-type injection site reactions, neutrophilic dermatosis-type injection site reactions, and systemic cutaneous reactions. Erythematous-type injection site reactions occur in approximately 40% of patients [6]. Although painful, these tend to resolve throughout treatment cycles and usually do not necessitate the discontinuation of the drug. Histopathology frequently shows lymphocyte infiltration. The last two patterns are uncommon and suggest type III immunologic drug reactions, manifesting with fever, which can be resolved through corticosteroid therapy, drug withdrawal, or both. With decitabine, respiratory and cutaneous reactions have been rarely reported. 

In cases where the permanent discontinuation of an HA is necessary, treatment alternatives are very limited.

Here, we present a case of atypical adverse reactions to two different HAs administered as monotherapy in a patient diagnosed with intermediate-risk MDS. 

## 2. Case Presentation

A 49-year-old patient was diagnosed with MDS with multilineage dysplasia following the detection of mild neutropenia (560/µL) after pneumonia. His only reported symptom was bone pain, localized to the lumbar region and lower limbs. He had a history of arterial hypertension, smoking (20 cigarettes/day), and psoriasis, previously treated with daily topical betamethasone and oral methotrexate 2.5 mg biweekly, subsequently replaced by ustekinumab 45 mg every 12 weeks, administered for 8 months and discontinued four months before the first Hematology consultation. Blood tests at the time of diagnosis showed mild anemia (hemoglobin 11.9 g/dL, reference range 13.0–17.0 g/dL), thrombocytopenia (66,000/µL, reference range 150,000–450,000/µL), and leukopenia (1620/µL, reference range 4000–11,000/µL), with neutropenia (1080/µL, reference range 1500–8000/µL), dacryocytes, erythroblasts, and 2% myeloblasts (reference range <5%). Bone marrow aspirate revealed 7% myeloblasts, and a trephine biopsy presented grade 1–2 fibrosis. Bone marrow karyotyping detected 47,XY,+14 [19]/46,XY [1], indicating trisomy 14 in 19 out of 20 metaphases. Next-Generation Sequencing (NGS) with a 30-gene myeloid panel showed no mutations. The patient was classified as having MDS with multilineage dysplasia and an intermediate-risk according to the IPSS-M.

**Figure 1:**
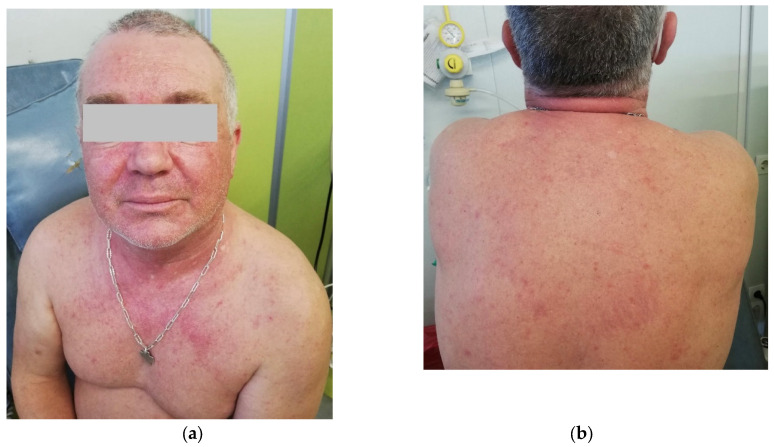
(**a**,**b**) Cutaneous erythematous, pruriginous, non-urticariform rash located on the face, thorax, abdomen, and inferior limbs.

A first-line treatment with AZA as a bridge for allo-HSCT was started, with a subcutaneous dose of 100 mg/m^2^ for 5 days in 28-day cycles, preceded by granisetron as an anti-emetic. After the 3rd day of the first cycle, the patient developed a cutaneous erythematous, pruritic, non-urticarial rash on the thorax, abdomen, and lower limbs, without mucosal involvement. Dexchlorpheniramine was administered, improving the symptoms, and a fourth dose of AZA was administered. Granisetron was substituted by metoclopramide to eliminate additional drugs with known allergenic potential. On the 5th day, the cutaneous rash worsened, including facial involvement, and AZA was administered after premedication with hydrocortisone and dexchlorpheniramine. No other symptoms, including respiratory, vascular, or gastrointestinal ones, were observed, nor was fever. On the 13th day, the patient complained of left-sided thoracic pain, cough, and wheezing. A thoracic X-ray did not show signs of respiratory infection, and the C-Reactive Protein levels were only mildly increased at 1.27 mg/dL (reference range <0.5 mg/dL). Azithromycin was given for five days, and 10 mg prednisone was continued until the next cycle. The second cycle was started with antihistamines and prednisone 20 mg as premedication. On the 1st day of the second cycle, cutaneous symptoms reappeared, with an erythematous rash on the trunk and back and facial edema (Figure 1). Symptoms improved with additional corticosteroids and antihistaminics. Blood tests excluded infection, and eosinophil counts were normal.

For this reason, AZA was withdrawn, and decitabine was started at a dose of 40 mg/day, preceded by methylprednisolone and famotidine. Between the 3rd and 4th days of the first cycle, the patient developed dyspnea and dry cough, leading to drug interruption. Later, on the 4th day, he developed a skin rash confined to the chest. Auscultation revealed crackles and wheezing. Blood tests and X-ray did not suggest infection. Respiratory syncytial virus, influenza A and B, SARS-CoV-2, and parainfluenza virus types 1 to 4 were not detected by a Polymerase Chain Reaction (PCR). Total serum IgE was measured and found to be within normal limits (91 IU/mL). Symptoms improved transiently with bronchodilators.

**Figure 2:**
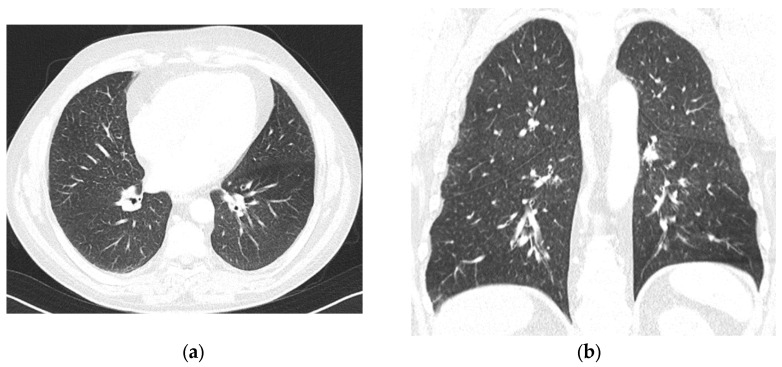
(**a**,**b**) Thoracic CT scan, after decitabine withdrawal and in the presence of respiratory symptoms, showing symmetric, diffuse lung involvement with nonspecific alterations and centrilobular alteration suggestive of small respiratory tract involvement.

Decitabine was withdrawn, and we assumed an adverse reaction to both medications. We decided to proceed directly to allo-HSCT. Meanwhile, the patient received intermittent pulses of prednisolone to manage persistent bone pain in the lower limbs, which had been present since the initial diagnosis. Despite Hb levels being > 11 g/dL, he started feeling tiredness two months after decitabine withdrawal. The patient was referred to the pulmonology department. A thoracic CT scan revealed symmetric, diffuse lung involvement with nonspecific interstitial findings and centrilobular changes suggestive of small airway involvement (Figure 2). A potential toxic hypersensitivity reaction with associated bronchiolitis was considered. Pulmonary function tests showed moderate bronchiolar and bronchial obstruction without hyperinflation, with a slight improvement (12%/260 mL increase in FEV1) following bronchodilator administration. Alveolar–capillary diffusion capacity (DLCO) was normal, and partial respiratory insufficiency was noted with a PaO2 of 71.1 mmHg. Treatment with inhaled corticosteroids, a long-acting muscarinic antagonist (LAMA), and a long-acting beta-agonist (LABA) was initiated and maintained. Respiratory bronchiolitis-associated interstitial lung disease (RB-ILD) and chronic obstructive pulmonary disease (COPD) were considered. Six months later, a follow-up thoracic CT scan showed stable diffuse interstitial and centrilobular changes.

Currently, the patient continues on 15 mg of daily prednisolone to manage persistent bone pain and is under ongoing monitoring while awaiting allo-HSCT. Recent bone marrow analysis showed normocellularity with a reduced myeloid-to-erythroid ratio due to erythroid hyperplasia, 5% CD34+ cells, and no significant fibrosis. Cytogenetic evaluation continues to reveal trisomy 14 in 4 out of 16 metaphases. The NGS panel remained unchanged from that at diagnosis.

## 3. Discussion

MDS confers morbidity and mortality and remains a challenge for physicians since there are few treatment options. In young, previously healthy patients with a considerable risk of progressing into acute myeloid leukemia, the only curative strategy is allo-HSCT. As a bridge to allo-HSCT and in patients not able to receive it, HAs are the only disease-modifying agents. Thus, it is of extreme importance that adverse events are effectively managed to avoid drug withdrawal.

Regarding cutaneous reactions, generalized skin toxicity to subcutaneous AZA is less common compared to local injection site inflammation. Y. Shimoda-Komatsu et al. [5] proposed a classification for cutaneous toxicity patterns after reviewing 17 cases from different authors and adding 4 cases from their own practice: erythematous-type injection site reactions with histological lymphocyte infiltration, neutrophilic dermatosis-type injection site reactions, and systemic cutaneous reactions.

Systemic cutaneous reactions vary between Sweet’s syndrome, neutrophilic panniculitis, urticarial rash, maculopapular erythematous eruption, and erythema annulare centrifugum, all presenting with fever. Neutrophilic dermatosis-type injection site reactions, despite local cutaneous involvement, also present with fever. Both patterns require treatment with corticosteroids, with some cases resolving after drug withdrawal only. In contrast, erythematous-type injection site reactions are better tolerated and allow for treatment continuation.

On the other hand, cutaneous reactions to decitabine are rare. Verma et al. described a case where, as expected, maculopapular lesions resolved after switching from azacitidine to decitabine in a 28-year-old patient with AML, with no reaction to the latter drug [6]. In contrast, in our case, the same cutaneous reaction reappeared after switching from azacitidine to decitabine, an unexpected outcome that suggests a possible shared underlying mechanism.

Pulmonary toxicity associated with systemic antineoplastic therapy is common, with various mechanisms proposed to induce it. However, this type of reaction is infrequent with HAs. Some pivotal phase III comparative studies (AZA 001, CALGB 9221) did not highlight interstitial pneumonitis or ILD, although AZA 001 reported a case of pulmonary fibrosis. Lung disease, in the form of interstitial lung disease (ILD) and pneumonitis induced by AZA, has been described in AML patients [2,3,4], all with different comorbidities contributing to respiratory frailty, such as age, COPD, and lipoid pneumonia related to cocaine use. All cases presented with respiratory failure, leading to AZA withdrawal, followed by corticosteroid treatment, with symptom improvement.

Similarly, there are rare cases of AZA-induced pneumonitis [7], interstitial and alveolar fibrosis [8], organizing pneumonia [9], and eosinophilic pneumonia [10] in MDS. All cases presented a late onset, fever, and respiratory failure. All corticosteroid-treated cases resolved after AZA withdrawal.

Even fewer cases of lung toxicity after decitabine are described: there was one case of idiopathic pulmonary fibrosis after 11 cycles of decitabine in a 89-year-old woman with AML, which resolved after corticosteroid therapy, allowing treatment to continue [11].

Our case differs from the ones previously reported for two main reasons. First, two different types of reactions—cutaneous and respiratory—occurred with the first hypomethylating agent, AZA. Second, similar reactions occurred in the opposing order—respiratory followed by cutaneous—with the second HA, decitabine, being accompanied by persistent respiratory complaints even after drug withdrawal and despite corticosteroid therapy. The clinical signs and symptoms also differed: while other cases often described fever and sudden respiratory failure, our patient experienced a slight cough and dyspnea progressing to chronicity. Furthermore, we cannot reliably diagnose ILD, pneumonitis, or any other inflammatory lung condition, as a lung biopsy could not be performed.

Given the temporal relationship between drug initiation and the onset of symptoms, we considered the skin and lung reactions to be related to hypomethylating agents. To clarify this hypothesis, we applied the Naranjo adverse drug reaction probability scale [12] for the four different events: cutaneous reaction after AZA, respiratory reaction after AZA, cutaneous reaction after decitabine, and respiratory reaction after decitabine. The scores are presented in Table 1.

According to this probability scale, the skin reaction was probably due to AZA and definitely due to decitabine, while respiratory toxicity was probably due to AZA and decitabine. Due to the patient’s smoking history, we cannot exclude COPD or R-BILD as the cause of prolonged respiratory symptoms. However, it is very unlikely that the first respiratory manifestations were due to this condition, since the onset coincided with HA administration and the patient had no prior symptoms. Smoking history could have facilitated the development of respiratory reactions after any chemotherapy agent.

Understanding the immunological mechanism of these manifestations remains challenging. They may be considered as type B drug reactions due to drug hypersensitivity, specifically of a delayed subtype. The late onset and low serum IgE levels suggest that the reaction was not IgE-mediated nor a pseudoallergy, as these reactions are typically immediate with high serum IgE levels. Therefore, we can exclude hypersensitivity pneumonitis and eosinophilic pneumonia. Furthermore, in the absence of hemolysis, new cytopenias, and fever, we can more confidently rule out type II or III hypersensitivity drug reactions. This leads us to believe that a type IV hypersensitivity reaction was the basis for the clinical symptoms [13,14,15]. 

## 4. Conclusions

To the best of our knowledge, this is the first case of respiratory and cutaneous reactions to two hypomethylating agents reported. Intolerance to this class of drugs impairs the use of the only disease-modifying treatment available for MDS. Although the majority of previously described cases resolved with accessible measures, the effective management of toxicities in scenarios like the one presented here is crucial to avoid compromising survival in this aggressive hematological malignancy.

## Figures and Tables

**Table 1:** The application of the Naranjo scale [12] to the suspected azacytidine and decitabine cutaneous and respiratory reactions.

	Cutaneous Reaction with AZA	Respiratory Reaction with AZA	Cutaneous Reaction with Decitabine	Respiratory Reaction with Decitabine
Are there previous conclusive reports on this reaction?	Yes, +1	Yes, +1	Yes, +1	Yes, +1
Did the adverse event appear after the suspected drug was administered?	Yes, +2	Yes, +2	Yes, +2	Yes, +2
Did the adverse event improve when the drug was discontinued or a specific antagonist was administered?	Yes, +1	Yes, +1	Yes, +1	No, 0
Did the adverse event reappear when the drug was readministered?	Yes, +2	Do Not Know ^a^, 0	Yes, +2	Do Not Know ^b^, 0
Are there alternative causes that could on their own have caused the reaction?	No, +2	No, +2	No, +2	No, +2
Did the reaction reappear when a placebo was given?	Do Not Know, 0	Do Not Know, 0	Do Not Know, 0	Do Not Know, 0
Was the drug detected in blood or other fluids in concentrations known to be toxic?	Do Not Know, 0	Do Not Know, 0	Do Not Know, 0	Do Not Know, 0
Was the reaction more severe when the dose was increased or less severe when the dose was decreased?	Do not Know, 0	Do Not Know, 0	Do Not Know, 0	Do Not Know, 0
Did the patient have a similar reaction to the same or similar drugs in any previous exposure?	No, 0	No, 0	Yes, +1	Yes, +1
Was the adverse event confirmed by any objective evidence?	Do not Know, 0	Yes, +1	Do not Know, 0	Yes, +1
Total score	8 = Probable	7 = Probable	9 = Definite	7 = Probable

Explanatory note: Scoring based on the Naranjo Adverse Drug Reaction Probability Scale—Each answer is assigned a specific score (e.g., +1, +2, 0 or -1), which may vary depending on the question, reflecting the likelihood of a causal relationship between the drug and the adverse event. A total score ≥9 indicates a ‘Definite’ reaction, 5-8 ‘Probable’, 1-4 ‘Possible’, and ≤0 ‘Doubtful’. a—respiratory symptoms started only after the last administration; b—there was no decitabine readministration, and respiratory symptoms persisted after the first try; AZA—azacitidine.

## Data Availability

The data presented in this study are available on request from the corresponding author. The data are not publicly available due to privacy restrictions.
